# Current status and progress of validation studies on the ICOPE scale: a scoping review

**DOI:** 10.3389/fpubh.2026.1817233

**Published:** 2026-06-09

**Authors:** Yuan Zhao, Mei He, Songye Zhao, Le Guo, Gan Li, Ying Tan, Jinjiao Yang, Binghua Lan, Yunpeng Su

**Affiliations:** 1 Department of Geriatrics, Dali University, Dali, Yunnan, China; 2 Department of Geriatrics, People’s Hospital of Dali Prefecture, Dali, Yunnan, China; 3 Department of Geriatrics, Affiliated Minzu Hospital, Hubei Minzu University, Enshi, Hubei, China; 4 Department of Health Management, Haiyuan College, Kunming Medical University, Kunming, Yunnan, China

**Keywords:** healthy aging, intrinsic capacity, older adults, screening tool, validation studies

## Abstract

**Background:**

With the rapid global population aging, systematically and early identifying and managing functional decline in older adults has become a core challenge in public health. The Integrated Care for Older People (ICOPE) framework and its screening tool, proposed by the World Health Organization (WHO), aim to prevent disability by assessing intrinsic capacity. However, there is a lack of systematic review of the implementation strategies, effects, and challenges of this tool in different global contexts. This study aimed to review the global implementation evidence of the ICOPE screening tool, analyze its integration paths and influencing factors, and provide a basis for constructing an integrated care model suitable for different resource environments.

**Methods:**

This study systematically retrieved and included literatures meeting the criteria, and comprehensively sorted and conducted comparative analyses of the design, population, positive screening rate, tool performance indicators (such as sensitivity and specificity), and implementation challenges of the included studies.

**Results:**

The study demonstrated significant heterogeneity in the global application of the ICOPE screening tool. The positive screening rates vary greatly due to differences in research design, target populations, and assessment criteria. The psychometric properties of the tool differ across various cultural backgrounds, and the implementation process faces common challenges such as disconnection between screening and intervention, uneven accessibility of digital tools, lack of evidence for long-term effects, and absence of unified diagnostic criteria.

**Conclusion:**

The ICOPE framework shows preliminary feasibility and global acceptance, but the performance of the screening tools needs to be interpreted in the context of specific cultures and populations. Future research should focus on unifying core measurement indicators, conducting long-term validity verification, and establishing a standardized implementation path integrating screening, assessment, and intervention to promote effective implementation of this strategy globally, especially in resource-limited areas.

## Highlights

- The effectiveness of the ICOPE screening tool varies significantly across regions in global applications.
- The distribution patterns of intrinsic capacity decline domains in older adults differ by region.
- The reliability and validity of the tool vary across different cultural backgrounds.
- The current implementation faces core challenges, such as disconnection between screening and intervention and lack of long-term evidence.
- Standardized assessment and integration pathways need to be established to achieve effective promotion.

## 1 Introduction

The world is aging quickly, and safeguarding the health and quality of life of older adults is a key area of concern in international public health. According to the World Health Organization (WHO), the global older population may increase from 1 billion in 2019 to 2.1 billion in 2050. In this context, a decline in intrinsic capacity(IC) (1) is known to contribute to higher rates of functional decline, hospitalization, and mortality risk among older adults. This situation has created a need for early detection and systematic interventions in the community (2). The traditional “disease-centered” model of health services is unable to meet the diverse and complex needs of older adults (3). WHO has established a framework of integrated care for older adults to support healthy aging. The framework aimed to sustain and improve IC and foster environments that are friendly toward older adults (4). The implementation of the framework depends upon a standardized screening tool, such as the integrated care for older people (ICOPE) screening tool. The tool is subjected to practical studies in countries, such as India, China, Brazil, Singapore, and France, to assess the practicality and initial effects in different sociocultural set-ups. A global “gold standard” to evaluate ICOPE has not been universally accepted. Many studies differ in measurement tools, evaluation criteria, and diagnostic thresholds, with significant differences in reliability, validity, and comparability (5). Presently, there are few studies on the effects of the ICOPE tool in terms of diagnosis (e.g., sensitivity and specificity), such as the Spanish VIMCI study. In contrast, several studies in China and Brazil focus on assessing implementation feasibility and early outcomes only, and not on an overall evaluation of the tool’s discriminant validity. Thus, there is an urgent need for more validation research of the tool’s psychometric properties to enhance its discrimination ability and usability across various populations and contexts. This study aimed to conduct a systematic review of the literature related to the implementation and validation of ICOPE globally from 2017 to 2026. Further, the review will also focus for advancements in validation and regional variation in screening and intervention related to “intrinsic capacity” in various areas. To systematically evaluate the implementation strategies and impacts of the ICOPE tool, this review may use the reach, effectiveness, adoption, implementation, and maintenance (RE-AIM) framework as its perspective. This framework facilitates a holistic evaluation of a tool’s coverage, effectiveness, and integration into diverse healthcare systems. Consequently, this study may provide a research-based framework for the formulation of a standardized and comparable system for implementing and evaluating ICOPE.

## 2 Materials and methods

### 2.1 Inclusion and exclusion criteria for literature

This study developed the following inclusion criteria for the literature. The study population should comprise community-dwelling older adults (aged 60 years or older). The study should address any aspects of implementation within the ICOPE framework, which can include, but are not limited to, screening, assessment, monitoring of decline, or intervention to enhance intrinsic capacity. Acceptable study types include original research studies, reviews, project reports, and conference abstracts, provided that the literature contains extractable original data or a clear explanation of the ICOPE implementation methods. Articles included in this study dated from 2017 to the present day. The published literature meeting the inclusion criteria may be systematically and manually searched. A manual and systematic review of the published literature meeting the exclusion criteria may be conducted to identify duplicate publications (see Table 1).

**Table 1:** Global ICOPE validation studies based on design, implementation, and key indicators.

No.	Country/region	Year published	Study design	Implementation phase	Sample size	Quantitative indicators	Implementation strategies; adaptation	Reference
1	France	2020–2021	Prospective cohort study	Large-scale implementation	10,903	Screening positive rate: 94.3%; vision impairment: 68.1%	Developed ICOPE app software and self-monitoring devices, offered freely on Apple and Android stores, enabling remote assessment and care reminders.	(6, 7)
2	China	2023	Cross-sectional diagnostic accuracy study	Strict validation	376	Prevalence of intrinsic capacity decline: 43%; screening positive rate: 69.1%	Led by Xuanwu Hospital, Capital Medical University, conducted the ICOPE-China project in communities, first validating the applicability of the WHO ICOPE screening tool in the Chinese population.	(10)
3	South Korea	2020	Randomized controlled trial	Strict validation	Not specified	The Korean Frailty Index for Primary Care (KFI_PC) already includes 8 core ICOPE items.	Implemented the ICOOP_Frail project in primary care clinics, integrating medical, social, and psychological support to person-centered enhance healthy aging.	(12)
4	Brazil	2025	Multicenter prospective cohort study	Large-scale implementation	3,838	Screening positive rate: 89.3%	Conducted the Projeto ICOPE Brasil across multiple regions, focusing on the Portuguese language localization and cultural adaptation validation of the ICOPE screening tool.	(14)
5	Mexico	2023	Prospective community assessment study	Pilot/feasibility study	387	Sensitivity: 0.82; specificity: 0.76	Conducted in Minatitlán, Veracruz; used mixed methods to evaluate the feasibility and acceptability of ICOPE at the community level.	(15)
6	Canada	2024	National multicenter prospective cohort study	Preparation stage	2,341	Intrinsic capacity decline detection rate: to be refined	Conducted in Ontario, British Columbia, and Quebec, aims to provide localized evidence for the nationwide promotion of ICOPE.	(19)
7	India	2022	Cross-sectional study	Strict validation	451	Men: 54.5%; high illiteracy rate	Conducted in the rural area of Jodhpur, Rajasthan (Thar Desert), validating the applicability of the ICOPE tool in populations with low education and in rural settings.	(11)
8	Vietnam	2023	Cross-sectional validation study	Pilot/feasibility study	200	Screening positive rate: 75.5%	Conducted in Ho Chi Minh City, evaluating the reliability, validity, and feasibility of the ICOPE screening tool in urban Vietnamese communities.	(13)
9	Qatar	2024	Mixed-method study	Pilot/feasibility study	1,248	Intrinsic capacity decline detection rate: To be refined	Used the RE-AIM implementation evaluation framework, combining quantitative and qualitative methods to assess implementation barriers and facilitators of ICOPE in the Middle East region.	(12)
10	Singapore	2024	Mixed-methods study	Preparation stage	367	Visual impairment: 42.0%	Conducted in a multicultural context, focusing on evaluating the acceptability and implementation strategies of ICOPE in multilingual, multicultural populations.	(16)
11	Kenya	2023	Descriptive case study	Preparation stage	Not specified	Data to be refined	Currently in the ICOPE implementation preparation stage, using case studies to map existing resources to provide a foundation for subsequent piloting.	(13)
12	Taiwan	2023	Prospective cohort study	Preparation stage	2,156	Cronbach’s α = 0.82	Conducted a prospective cohort study in the community to validate the reliability of the ICOPE screening tool, providing localized evidence for regional promotion.	(17)
13	Hong Kong	2023	Prospective cohort study	Preparation stage	1,847	Screening positive rate: 72.1%	Conducted in Shui Po, Yau Tsim Mong, and Kowloon City districts, leveraging 12 older adults district centers and 8 neighborhood older adults centers to assess the implementation effectiveness of ICOPE in urban communities.	(18)
14	Spain	2022	Cross-sectional diagnostic accuracy study	Pilot/feasibility study	207	Sensitivity: 0.438–0.889; specificity: 0.682–0.96	The VIMCI cohort study validated the diagnostic accuracy of the ICOPE screening tool in the Spanish population, providing a basis for subsequent large-scale implementation.	(8)
15	Italy	2023	Longitudinal cohort study	Pilot/feasibility study	400 + (aged 75+)	Intrinsic capacity decline detection rate: to be refined	Applied the ICOPE model in primary care, combined with tools like the frailty screening test and chair rise test, to evaluate the feasibility and effectiveness of ICOPE within the Italian healthcare system.	(9)

### 2.2 Search strategy

Databases in both English and Chinese, including CNKI, Wanfang Database, VIP Database, PubMed, Web of Science, Embase, Scopus, and SinoMed, were accessed through computer searches. The databases for official and grey literature encompassed WHO IRIS, the Global Health Library, and the websites of health departments from various nations. The search timeframe was set from January 2017 to January 2026 to include significant research conducted since the ICOPE framework was officially launched by WHO. The search strategy integrated subject terms alongside free text and used the Boolean logic operators for combinations. For instance, in CNKI, the Chinese search equation was structured as follows: (SU = ‘WHO ICOPE’ + ‘Integrated care for older adults’ + ‘ICOPE’) AND (SU = ‘Intrinsic capacity’ + ‘Intrinsic function’ + ‘IC capacity’) AND (SU = ‘older adults’ + ‘Old age’ + ‘Advanced age’) AND (SU = ‘Screening’ + ‘Assessment’ + ‘Implementation’ + ‘Pilot’ + ‘Validation’ + ‘Tool’). Taking PubMed as an example, the English search formula is as follows: (“ICOPE”[Title/Abstract] OR “Integrated Care for Older People”[Title/Abstract] OR “Integrated Care for Older Persons”[Title/Abstract]) AND (“Intrinsic Capacity”[Title/Abstract] OR “IC Capacity”[Title/Abstract]) AND (“Aged”[MeSH Terms] OR “Older adults”[Title/Abstract] OR “Older Adult”[Title/Abstract]) AND (“Screening”[Title/Abstract] OR “Implementation”[Title/Abstract] OR “Validation”[Title/Abstract] OR “Pilot”[Title/Abstract] OR “Instrument”[Title/Abstract]).

### 2.3 Literature screening and data extraction

The EndNote application was used for the collection of literature and deletion of duplicates in this study. Two researchers screened the literature. After analyzing the literature, a classification was developed to form a comprehensive and impartial evaluation index system. In situations where agreement was not reached during screening; a third party was involved in discussions until agreement was reached. When needed, consensus meetings or a third, senior reviewer (the corresponding author) resolved disagreements. During data extraction, the collected information includes important evaluation indicators, including the country or region of the study, type of study, publication year, study design, sample size at the implementation stage, positive screening rate, intervention coverage rate, sensitivity and specificity of the tool, and the detection rate of decline in intrinsic capacity.

## 3 Results

### 3.1 Study selection and global overview of key indicators

A preliminary review of articles yielded a total of 2,764 articles. The screening was done rigorously using the inclusion and exclusion criteria on the titles, abstracts, and full-texts. Ultimately, 14 essential articles that satisfied the criteria were included in this evidence-based guideline. The publication distribution of these articles included Europe (France (6, 7) (*n* = 2), Spain (*n =* 1) (8), Italy (*n =* 1) (9), and Andorra (*n =* 1) (6)); Asia (China (*n =* 1) (10), India (*n =* 1) (11), South Korea (*n =* 1) (12), Vietnam (*n =* 1) (13), and Qatar (*n =* 10) (12)); America (Brazil (*n =* 1) (14) and Mexico (*n =* 1) (15)); Africa (Kenya), Singapore (16), Taiwan (17), Hong Kong (18), and Canada (19). Most studies (*n* = 9) included in articles published between 2017 and 2026 were published after 2022.

**Figure 1:**
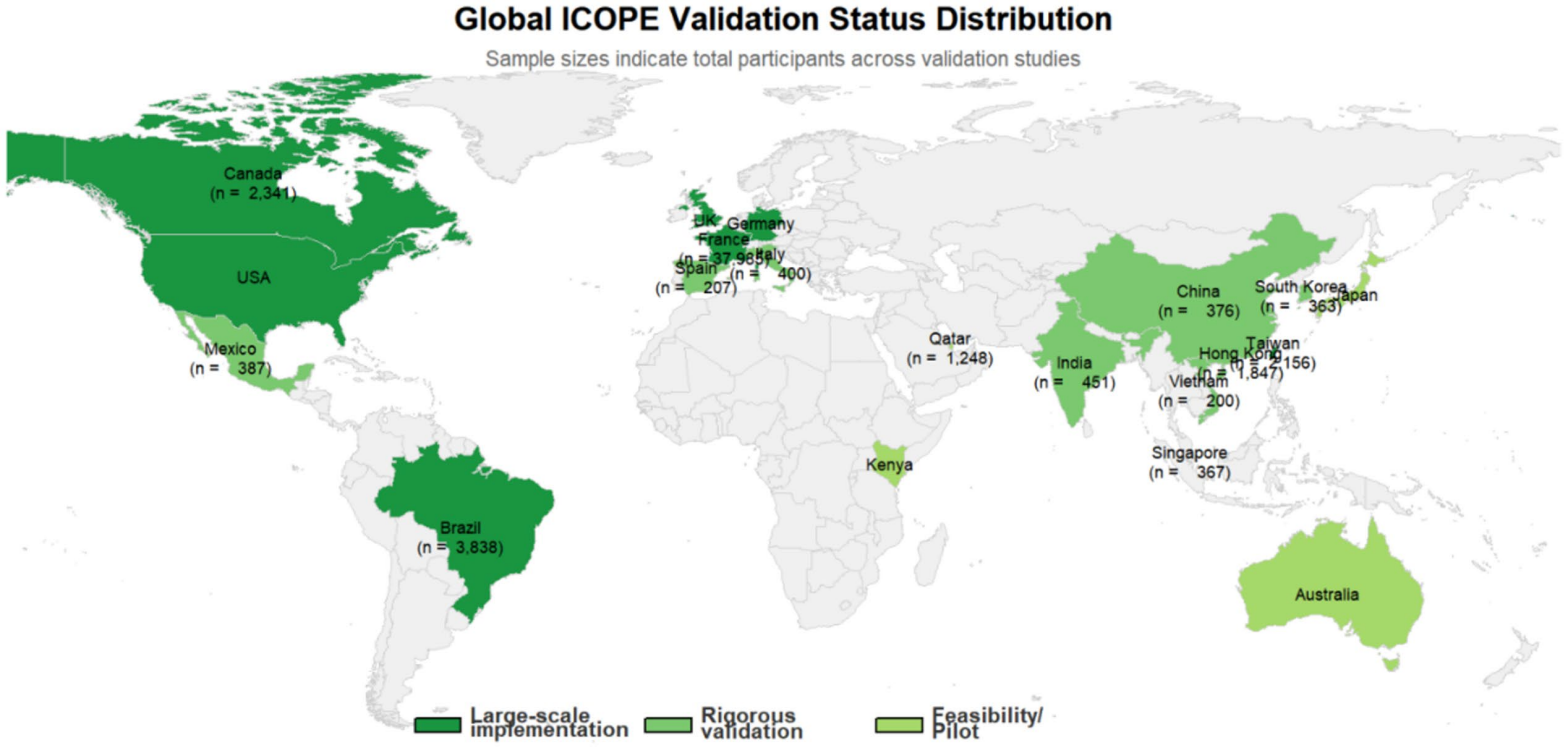
The distribution situation of each country is detailed in the following.

**Figure 2:**
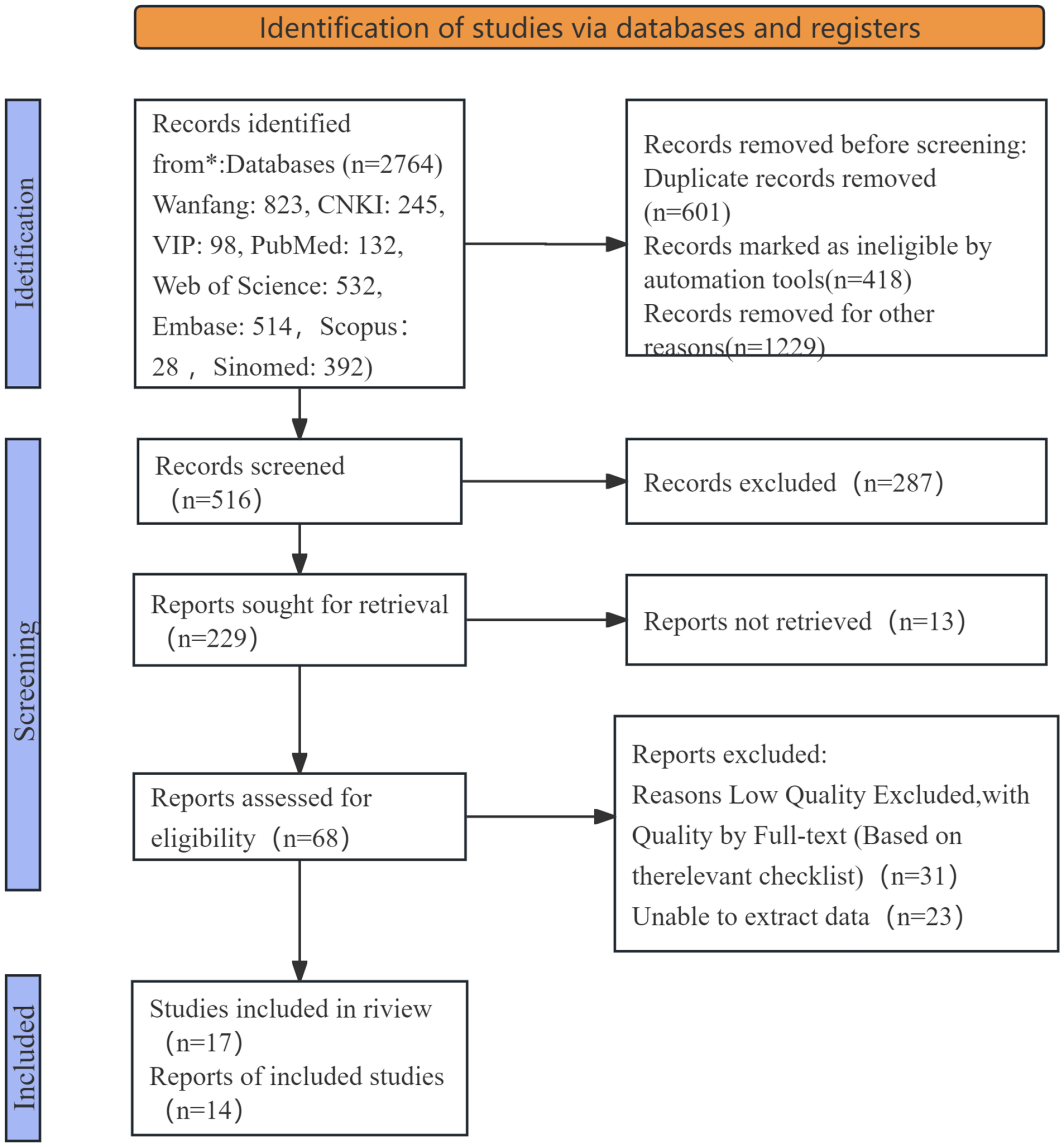
Screening flowchart.

Research methodology and participant characteristics differ widely across global use of the ICOPE screening tool. The first screening of published articles identified 2,764 studies. The titles and abstracts, along with the full texts, were screened rigorously according to the inclusion and exclusion criteria. Ultimately, 14 essential articles that met the inclusion criteria were included in this guideline. Figures 1, 2 shows a PRISMA flow diagram depicting the selection of studies. The illustration shows the sample sizes of ICOPE validation studies across different regions worldwide from 2021 to 2025. The varying shades of green on the map indicate sample size, with the deepest green indicating the highest sample size. The main tools used for measurement were the WHO ICOPE Screening Tool and its modifications. The number of participants varied, a lot from hundreds to tens of thousands. Presently, the most important project in France is INSPIRE with several participants (*n* = 27,082). The key performance indicators show significant differences. The sensitivity of the ICOPE screening tools ranges from 0.76 to 0.89; the specificity ranges from 0.69 to 0.76; and a large disparity in positive screening rates from 40 to 94.3%. Preliminary diagnostic accuracy results from Spain show a sensitivity of 0.889 for the cognitive domain, while sensitivity for most functional domains ranged from 0.438 to 0.569, and specificity from 0.682 to 0.96. These deviations indicate the substantial risk of false positives. The subsequent analysis of deviation is based on these results.

### 3.2 Structured comprehensive analysis: determinants of heterogeneity

In an effort to go beyond descriptive statistics, this study structured the results to identify the underlying causes of variation. The three related factors include development of regional implementation, different research purposes, and varying methodological benchmarks.

#### 3.2.1 Geographic region and implementation stage: the role of infrastructure

A unique developmental gradient is shaped by differences in digital infrastructure and the maturity of health systems. From a geographical perspective, large-scale validation and deployment are underway in Europe (particularly France and Spain) and in several Asian nations, including China and South Korea. This progress has been facilitated by the comprehensive embedding of digital solutions. Initiatives in France and China encourage the use of digital tools (e.g., ICOPE monitor and chatbots) to increase screening coverage; in France, approximately 56% of screenings are conducted through this application. Another implementation strategy must be sought in regions that lack such infrastructure. Regions in Latin America, parts of Africa, and some parts of Southeast Asia are still in the pilot and early project stages, with a focus on basic feasibility studies. A study in Mexico focused on community-level feasibility, whereas studies in Kenya and Canada are currently in the preparatory phase. The “implementation stage” variation is not about timing but local capability to support digital and multi-disciplinary integration.

#### 3.2.2 Research design and tool performance: the trade-off between reach and precision

The observed range of positivity rates is primarily an artifact of study design objectives, yielding a trade-off between reach and precision. The INSPIRE project (in France) and another study in Brazil are the two large implementation studies. Research emphasizing “reach” may produce high screening rates but low precision. The positive screening rate in the INSPIRE project (France) was 94.3%. Although the screening yielded extensive coverage, it implies a high risk of false positives, which could strain the healthcare system. On the other hand, diagnostic accuracy studies are otherwise conducted with psychometric rigor, revealing the important limitation of the tool’s sensitivity in non-cognitive domains. The sensitivity in the cognitive domain was at least 0.889. Furthermore, across most functional domains, sensitivity ranged from 0.438 to 0.569, in the VIMCI study (Spain). The discrepancy indicates uneven detection across domains and also suggests that indiscriminate implementation in primary care may miss declines in mobility or sensory domains.

#### 3.2.3 Cultural adaptation and methodological inconsistency: barriers to comparability

The lack of benchmarks for culture adaptation and assessment has introduced systematic biases in the data, posing the biggest challenge in achieving global comparability. In the first place, cultural and linguistic factors affect the performance of specific products. Validation efforts conducted in non-Western contexts demonstrate that extensive localization is required. For example, an Indian study focused on a rural population with limited education, while a Brazilian initiative validated the Portuguese-language version of the tool. Subsequently, direct cross-cultural comparisons of screening rates are methodologically limited, as the psychometric properties of the tools differ across cultures. Furthermore, varying assessment criteria also lead to fragmentation of the data. At present, there are no standard definitions for what constitutes a “positive” screening result. A research effort in Brazil implemented ICOPE with IVCF-20, and a study in Mexico used MUSIQ to evaluate the implementation environment. The “screening-assessment” process varies widely because a diverse range of additional instruments is used. Due to these different methodologies, it is impossible to compare prognostic values among studies, and the findings may not be globally applicable.

## 4 Discussion

### 4.1 Global implementation overview and characteristics of positive screening rates

This review provides the first systematic synthesis of the heterogeneous patterns in the global validation of the ICOPE tool. By applying the RE-AIM framework (41), three core mechanisms underlying this heterogeneity emerge: disparities in implementation stages driven by infrastructure, the trade-off between reach and precision in study design, and the profound impact on cultural adaptation. These findings provide a direct evidence-based foundation for constructing standardized implementation pathways tailored to different resource settings. To investigate the global results using a systematic implementation approach, this study first assessed the ICOPE screening tool’s reach and coverage. Studies from different parts of the world show that the ICOPE framework, which the WHO recommends to enhance and maintain intrinsic capacity, is a key strategy to support healthy aging. The real rates of positive cases vary greatly across countries. One such study, the INSPIRE study in France, screened 27,082 older persons, achieved broad coverage. Other studies reported sample sizes that ranged from hundreds to thousands. The differences not only indicate the dispersion of health status among older adults but are also largely influenced by factors such as study design, target population characteristics, evaluation criteria, and socio-cultural contexts. In a thorough community screening effort in France, 90.8% of first-round participants tested positive during the preliminary evaluation. This indicates that older people in this country, not just diagnosed ones, but also non-healthcare professionals and self-reported ones, are at risk of declining intrinsic capacity. In contrast, a study of mainly independent older people in Catalonia, Spain, found that while 79% experienced a decline in at least one domain, pilot screening in this region was more targeted and generally followed stricter diagnostic criteria. The national ICOPE assessment for Taiwan in 2023 covered a more extensive community population aged 65 and older, including indigenous people aged 55 and older. The rates of detection were low with 15.75% for cognitive impairment, 15.52% for vision, and 4.15% for mental health issues. This difference arises primarily from the different scopes of the screenings. When testing is directed at a broader community rather than a defined high-risk group, the positivity rate is expected to be lower.

### 4.2 Evaluation of implementation outcomes and ongoing issues

Going beyond coverage, the problems related to efficiency and feasibility in the implementation process are serious. The implementation of ICOPE faces many barriers. Significant difference between screening and screening intervention for stroke. The majority of the research is on screening, and no data are available on what happens afterward and how far the intervention leads; thus, the effect of ICOPE on preserving function and reducing mortality cannot be assessed. A study in Brazil found that people with multi-domain deficits are at a higher risk of death than people with a deficit in one domain. Still, data on the interventions remain incomplete. Furthermore, there is a technological gap regarding digital solutions. The ICOPE MONITOR App and ICOPE BOT in France have significantly improved the efficiency of screenings, yet the self-reported functional abilities of the population seem much better than the assessments made by health professionals. This is a clear example of a “digital divide” that may lead to missed diagnoses of high-risk individuals. Research conducted in Singapore and Mexico has shown that older adults are not very accepting of digital tools. Another factor detracting from the overall management is the lack of sufficient interdisciplinary collaboration and incomplete referral pathways. For instance, the need to establish pathways for referrals to optometry and ENT specialists was highlighted in Singapore. No criteria have been established to determine the standards of adjustment for adaptation and localization of culture. A Brazilian study applied ICOPE with IVCF-20. At the same time, research in Mexico used MUSIQ to map the implementation environment, which displayed a lack of support in the external environment for effective implementation. France’s attainment of 100% intervention coverage offers a model that could help with these issues. It is important to enhance the training of personnel in primary healthcare within a collaborative framework that incorporates different disciplines. This study further localize the tools so that they fit the local healthcare system and context rather than simply being translated directly. Thus, validation studies regarding the ICOPE scale as a screening tool of the framework are witnessing a growing trend of diversifying in recent years with regard to the improvement of standardization, validation of cross-cultural applicability, and broadening of predictive validity. The latest evidence shows that the ICOPE scale may detect functional decline in older people with respect to cognition, mobility, and psychological wellbeing through a two-step assessment (screening followed by a comprehensive assessment). The ICOPE scale showed a sensitivity of 95% and specificity of 57.6% (20); so, item design should be further improved as per local needs.

The ICOPE scale incorporates three technological advancements. First, it evaluates several dimensions, overcoming the limitations of the one-dimensional studies conducted previously. The evaluation of integrated care across multiple domains may assist in developing a better understanding of the global health of older people, through the independent evaluation of cognition, mobility, sensation, vitality, psyche, and vision (21). For example, a randomized controlled trial in Chaoyang District, Beijing, found that people in the ICOPE intervention group, compared with those in the control group, had an improvement in mobility (SPPB scores) and psychological wellbeing (GDS scores) after 6 months (*p* < 0.05) (22). In addition, the design is easy to use for primary care. A validation study in Taiwan found that the brief screening was completed in 2 min and was highly associated with the full MMSE (*r* = −0.752) (23). Lastly, it was quite extraordinary to foresee the clinical outcomes of a negative event. According to a Brazilian study, scores in the mobility and hearing domains presented a significant association with falls (OR = 2.27, *p* = 0.0157) (24). Similarly, the French MAPT study demonstrated its ability to predict disability over 5 years (aHR = 1.74) (25). At present, the research focuses on localization and new technologies. Researchers conducted a receiver operating characteristic curve analysis in Shanghai, discovering a threshold effect (an inflection point value of 4.476) for metabolic markers TyG–WHtR and a decline in the vitality domain (26). It is well-established that biomarkers may be used alongside the ICOPE Scale. A research study using a structural equation model conducted in Hong Kong indicates that ICOPE scores were significantly related to self-care abilities (*β* = 0.21) and social participation (*β* = 0.31) (27). The importance of these studies must be kept in mind while assessing social functioning. Moreover, in the context of the INSPIRE-T cohort study, a dynamic prediction model for the ICOPE scale was developed by merging multiple data types (clinical assessments, biological samples, and digital monitoring) (28). This shows that validation research is changing from static screening methods to dynamic monitoring methods.

### 4.3 Analysis of the implementation path of ICOPE as an integrated care strategy

For the ICOPE framework to function optimally, the screening tools must be accurate and enable a smooth transition from screening to intervention. Lessons from international policies have identified three shared models of implementation that highlight critical issues on the roadmap to integration and local adaptation strategies. The French project INSPIRE had already screened 27,000 older adults through the digital tools such as ICOPE MONITOR. Approximately 56% of the screening was conducted through this app, demonstrating the potential of digital technology for outreach (6, 7). However, findings reveal a significant threat posed by the gap between front-end and back-end integration. Despite a positive screening test rate of 94.3%, only 8.9% of participants underwent a second test. The rapid rollout of digital screening initiatives, coupled with limited professional capacity for assessment, constitutes the main barrier in the “screening-assessment” stage of the integrated care pathway.

In contrast, the Brazilian Proyecto ICOPE Brasil adopts a collaborative approach to localization rather than a screening volume approach. This project may ensure the Portuguese localization and cultural fit of ICOPE screening instruments while exploring models of multi-disciplinary collaboration. Within the context of existing community healthcare, this method emphasizes the establishment of an integrated pathway through personnel training and inter-departmental links. Coverage appears to advance slowly at first; however, it is heavily nuanced and impacted by a focus on sustainable services and groups.

The third model is similar to the ICOPE–China, but implemented within China. This model relies on major medical organizations, such as Xuanwu Medical College Hospital, to conduct verification and community applications, thereby establishing a direct link between experts and the grassroots level (10). The differences between these models signal varying dependencies in the design of integrated care pathways. The focus of the studies is on the kind of collaboration taking place among personnel (professional team and local staff) and their interaction in exchanging information (through automated digital systems or manual referrals). Moreover, these studies investigate service–delivery integration, ranging from standard procedures to flexible approaches, essentially reflecting the democratic or participatory nature of the process.

Future integration must adopt a comprehensive approach. This involves using digital resources to enhance the initial “screening” process while simultaneously developing a robust “assessment-intervention” support framework on the backend, akin to established referral pathways and collaborative teams spanning multiple disciplines. It is crucial to align these pathways with local healthcare facilities and relevant cultural insights.

### 4.4 Analysis of sources of heterogeneity

The differences in positive screening rates observed across the included studies are likely due to fundamental differences in objectives, population characteristics, and methodology. The studies do not necessarily reflect differences in health status per se.

#### 4.4.1 Design-induced variability

The vastly different positivity rates, ranging from 94.3% in the French INSPIRE study to 15.75% for cognitive impairment in the Taiwan assessment, primarily reflect differences in the aims of the study designs. The French trial was one of large-scale implementation cohorts. A screening approach was used to detect any risk of decline. This approach yields high sensitivity but carries a high risk of false positives. In contrast, other studies that targeted specific high-risk populations or applied stricter diagnostic criteria, this study resulted in a low positivity rate. This phenomenon, known as “dilution effect,” shows that higher positive rates in the implementation studies may suggest over-screening or a need for tighter filters for secondary assessment rather than intrinsic capacity decline being more prevalent.

#### 4.4.2 Psychometric and cultural variability

The variability is further complicated by the tool’s inconsistent psychometric efficacy across different cultures. Validation research indicates significant fluctuations in sensitivity; for instance, the Spanish VIMCI study demonstrated high sensitivity (≥0.889) within the cognitive domain, whereas majority of the functional domains exhibited sensitivity levels ranging from 0.438 to 0.569. This suggests that the tool’s capacity to identify decline is inconsistent across various domains and populations. Additionally, the absence of standardized evaluation criteria for areas such as “vitality” and “mental state” results in differing thresholds for a positive screening outcome. Cultural adaptation is also crucial; for example, a study conducted in Brazil specifically aimed at validating the Portuguese localization of the tool, underscoring that both linguistic and cultural factors directly influence screening results.

#### 4.4.3 Implementation context

Finally, the context of implementation varies. The gap between screening and intervention in many studies means that the measured rate of positive screenings rarely leads to a verified diagnosis. This complicates the accurate calculation of true prevalence.

## 5 The limitations of the study and the directions for future research

The ICOPE scale developed by the WHO has been found to be sensitive and pragmatic for assessing decline in intrinsic capacity (IC) among older adults (29). However, although a formal risk of bias assessment is not always requisite for scoping reviews, it is essential to acknowledge the variability in study quality among the included studies and has potential impact on the interpretation of findings. Important limitations in the validation studies still exist and must be addressed urgently.

First, the specificity of the scale tends to vary, producing noticeable irregularities in sensory (such as vision) and cognitive areas. For example, Giudici et al. (30) reported a specificity of 2.7% for vision screening and 55.6% for cognitive factors. This indicates that the scale may produce a large number of false-positive results, which can cause overuse of full evaluations and misappropriation of healthcare resources. On the other hand, vitality only has a sensitivity of 51.3% (31), failing to address issues such as malnutrition. Furthermore, the scale emphasizes constraints in adapting to different cultures or physiologies.

Second, the samples used in the validation studies are often unrepresentative. At present, the research on older adults has been conducted primarily among community-dwelling older adults, which is on a particular urban site (e.g., Beijing, Hong Kong, and Shanghai), with average age of 84 years. This focus excludes younger older adults and those living in rural areas, limiting the generalizability of the validation results to broader, more diverse populations.

Further, majority of the studies are cross-sectional in nature (25), resulting in a lack of longitudinal data needed to assess the predictive validity of the scale, more specifically, its long-term capacity to serve as an early indicator of falls (32) or cognitive decline (33). Moreover, a significant lack of standardization, and challenges in adaptability to culturally specific issues are evident. The ICOPE scale items (e.g., “date of the week”) may be subject to environmental factors, including language and education (22), and the criteria for “vitality” or “mental state” assessment (e.g., MNA–SF and GDS scales) vary between regions (34). This variability makes direct comparison of the findings with other studies and restricts the global applicability of the scale (35).

In conclusion, the comorbid conditions were not assessed for their multidimensional interactions. The ICOPE scale consists of six different IC domains, but it does not examine whether they can be synergistic or antagonistic (36). The associations between decreased vitality, metabolic-inflammatory markers (e.g., NLR and TyG index), and association between declined locomotor and cognitive functions (37) underscore the need for more sophisticated models to enhance screening.

Future research should investigate the existing methodological diversity that may support the ICOPE transition from varied validation approaches to standardized applications. Standardizing evaluation criteria and screening instruments must be the primary objective. For parameters such as “vitality” and “mental-wellbeing,” this study should advocate for standardized tools with cross-cultural validity rather than different scales such as MNA-SF and GDS. Developing a set of core outcomes based on global or regional consensus would allow researchers to compare patterns of functional decline among older people across countries. The emphasis should be on capturing key dimensions (such as functional status, quality of life, rates of service use, and costs) rather than focusing solely on the rates of positive screening, which are difficult to verify. The proposed outcome measures will also facilitate resource planning (22).

In view of this methodological agreement, research designs should be more systematic and cohesive. On the one hand, there is a need to identify core outcome indicators (such as functional status, quality of life, and mortality) through multi-center cohort studies. Additionally, biomarkers and dynamic threshold tools (38) should be integrated to improve the accuracy and generalizability of measurements. Concurrently, the ICOPE framework must be incorporated into primary healthcare and long-term follow-up systems, and cost-effective analyses must be conducted. Attention should also be paid to implementation in low-income countries, and the application of ICOPE in low-resource settings requires further examination. There is a strong need to increase the multidisciplinary collaboration and systematic training for better quality implementation. The impacts on target populations, especially high-risk target populations such as long-term care home residents and older adults in rural areas, should be monitored. In addition, to further develop a more comprehensive evidence base aimed at promoting informed policy development, the cost-effectiveness of ICOPE in tiered diagnosis and treatment should be assessed (39). The ultimate focus is to build a framework for evidence-based, cost-effective integrated care that uses local strength and is sensitive to local resources.

Given this methodological consensus, research designs should be more systematic and cohesive. First, outcome measures such as function, quality of life, and survival should be determined using a multi-center cohort study. Moreover, to improve the precision and generalizability of the measurements, biomarkers and dynamic threshold tools may be used (40). In contrary, the application of the ICOPE framework in primary healthcare, follow-up system in long-term, and cost-effectiveness analysis should be priorities. Additionally, research must investigate the implementation of ICOPE in low-income countries and its potential use in low-resource settings. Collaborations between disciplines and systematic training are needed for a better quality of implementation. Effects on target groups, in particular, vulnerable populations such as long-term care home residents and older adults living in rural areas, should be monitored closely. A strong need for cost-effective evaluations of ICOPE in stratified diagnosis and treatment to establish stronger evidence base for informed policy-making (39). This study aimed to develop a model of integrated care that is evidence-based, cost-effective, draws on local strengths, and is sensitive to local resources.

## 6 Policy and practical implications

At the policy level, the first step is to embed ICOPE into the national primary healthcare system. National initiatives such as China’s “Healthy Aging 2030” and Brazil’s Sistema Único de Saúde (SUS) may facilitate the integration of ICOPE, enabling early detection, comprehensive management, and the development of referral pathways from community to specialist care. Also, a network of collaboration through various disciplines, which has established referral channels, is necessary to ensure that these people receive treatment promptly after screening positive. An exemplary model is the French initiative, which trained non-specialist personnel (e.g., nurses and pharmacists) to conduct screening, thereby alleviating the workload of specialist physicians. Furthermore, it is important to strengthen the training of primary healthcare providers to improve their capacity to deliver ICOPE and to ensure the integrity of the screening and assessment process. Finally, to ensure inclusivity among different groups, it is also necessary to address the digital divide. Additionally, a mixed bag (such as digital and manual) should be used to meet the needs of different groups.

## 7 Conclusion

This study gathers global data affirming the validation of the WHO ICOPE screening tool, albeit with considerable variation in implementation and effect across different cultures and regions. Healthcare institutions must prioritize creating unified implementation tactics that are adapted to local assets to address these issues, as the system that validates the process does not yet provide a cohesive diagnostic standard. Based on these findings, this study recommend three actionable steps. First, future studies must focus on harmonizing key measurement indicators, since the psychometric characteristics of the tool vary across different contexts and there are no common evaluation criteria. In addition, there is a need to develop a closed-loop management framework that links screening, assessment, and intervention due to the existing disjuncture between screening and intervention. A mixed model, digital and traditional, should be implemented to ensure equity and address the digital divide. Thus, reliance on digital tools should not result in omissions of diagnosis for a considerable section of high-risk groups. Combating fragmentation requires collaboration across disciplines and the establishment of clear referral pathways.

## Data Availability

The datasets presented in this article are not readily available due to license restrictions. Further inquiries can be directed to the corresponding authors.
